# Pattern matching through Chaos Game Representation: bridging numerical and discrete data structures for biological sequence analysis

**DOI:** 10.1186/1748-7188-7-10

**Published:** 2012-05-02

**Authors:** Susana Vinga, Alexandra M Carvalho, Alexandre P Francisco, Luís MS Russo, Jonas S Almeida

**Affiliations:** 1 Instituto de Engenharia de Sistemas e Computadores: Investigação e Desenvolvimento (INESC-ID), R. Alves Redol 9, 1000-029 Lisboa, Portugal; 2 Dept Bioestatística e Informática, Faculdade de Ciências Médicas - Universidade Nova de Lisboa (FCM/UNL), C. Mártires Pátria 130, 1169-056 Lisboa, Portugal; 3 Instituto de Telecomunicações (IT), Av. Rovisco Pais 1, Torre Norte, Piso 10, 1049-001 Lisboa, Portugal; 4 Instituto Superior Técnico, Universidade Técnica de Lisboa (IST/UTL), Av. Rovisco Pais 1, 1049-001 Lisboa, Portugal; 5 Div Informatics, Dept Pathology, University of Alabama at Birmingham, USA

## Abstract

**Background:**

Chaos Game Representation (CGR) is an iterated function that bijectively maps discrete sequences into a continuous domain. As a result, discrete sequences can be object of statistical and topological analyses otherwise reserved to numerical systems. Characteristically, CGR coordinates of substrings sharing an *L*-long suffix will be located within 2*^-L ^*distance of each other. In the two decades since its original proposal, CGR has been generalized beyond its original focus on genomic sequences and has been successfully applied to a wide range of problems in bioinformatics. This report explores the possibility that it can be further extended to approach algorithms that rely on discrete, graph-based representations.

**Results:**

The exploratory analysis described here consisted of selecting foundational string problems and refactoring them using CGR-based algorithms. We found that CGR can take the role of suffix trees and emulate sophisticated string algorithms, efficiently solving exact and approximate string matching problems such as finding all palindromes and tandem repeats, and matching with mismatches. The common feature of these problems is that they use longest common extension (LCE) queries as subtasks of their procedures, which we show to have a constant time solution with CGR. Additionally, we show that CGR can be used as a rolling hash function within the Rabin-Karp algorithm.

**Conclusions:**

The analysis of biological sequences relies on algorithmic foundations facing mounting challenges, both logistic (performance) and analytical (lack of unifying mathematical framework). CGR is found to provide the latter and to promise the former: graph-based data structures for sequence analysis operations are entailed by numerical-based data structures produced by CGR maps, providing a unifying analytical framework for a diversity of pattern matching problems.

## Background

Biological sequence analysis remains a central problem in bioinformatics, boosted in recent years by the emergence of new generation sequencing high-throughput techniques [1,2]. With the advances of new technologies, the need for efficient algorithms for string representation and processing is increasing continuously.

Sequence representation plays a particular role in this regard, being the first step that both enables and conditions subsequent data processing, and has been the object of several analytical approaches over the last years [3]. The development of string processing algorithms based on different data structures, particularly suffix trees and suffix arrays, has been increasing steadily [1,4]. The classical version of these structures enhances the functionality of the underlying sequence by efficiently supporting several look-up procedures [4]. In particular, these structures can be used to locate substrings within the index sequence, which in turn yields efficient algorithms for complex problems such as the longest common substring, initially thought to have no linear time solution. Unfortunately, the speed up obtained from these structures comes with an heavy memory cost. This cost has been extensively researched, the most successful trend consisting of using data compression techniques, such as Lempel-Ziv data compression, Burrows-Wheeler transform, and local compression methods, achieving dramatic improvements in efficiency (see [5] for a survey). Moreover, these theoretical results allowed for the development of compressed aligners that have significantly reduced the resources necessary for sequence alignment [6-8], namely for aligning reads from pyro-sequencing platforms [9].

In parallel to these efforts, a particular prolific technique at engendering novel sequence analysis algorithms is the *Chaos Game Representation *(CGR), based on *Iterated Function Systems *(IFS), firstly proposed more than two decades ago [10]. This compact, lossless and computationally efficient representation allows the visualization of biological sequences and patterns, with a convenient visual appearance. In fact, CGR captures the patterns arising from distinct *L*-tuple compositions in DNA sequences [11], enabling whole genome representation and visualization [12]. It was also shown that CGR represents a generalization of *genomic signatures *[13,14], allowing to characterize different species by associating them with distinctive sequence statistics [12].

In addition, CGR can be interpreted as a generalization of Markov Chain models [15] and provides tools for the alignment-free analysis and comparison of biosequences [16], namely for DNA entropy estimation [17,18]. Some other applications of CGR include the problem of HIV-1 subtype classification [19], detection of persistent dynamic of human spike trains [20], horizontal transfer detection [21], phylogeny and extraction of representative motifs in regulation sequences [22], to name a few.

Although CGR has been successfully applied to a wide range of problems, its strength for general string matching problems is, so far, mostly unexplored. In this work we propose CGR as a solution for exact and approximate string matching problems, showing that this representation might bring useful algorithmic tools for biological sequence analysis and more general applications.

## Methods

We start this section by introducing some notation and recalling the definition and relevant properties concerning CGR. Thereafter, we embed CGR maps in the Cantor set in order to elicit longest common suffixes in an efficient and unambiguous way. As we shall see in the Results section this enables the retrieval of longest common extension queries in constant time, which in turn yield efficient algorithms for more complex string matching problems.

Let *S *denote a string of size *N*. In all what follows, *S*[*i*] represents the *i*'th symbol of *S*, with 1 ≤ *i *≤ *N*. Each *S*[*i*] is a DNA symbol representing each nucleotide or base (*A, C, G, T*), i.e., *S*[*i*] ∈ Σ = {*A, C, G, T*}. Moreover, *S*[*i .. j*] is the *substring *of *S *that starts at the *i*'th and ends at the *j*'th positions of *S*, with 1 ≤ *i *≤ *j *≤ *N*. The *length *of substring *S*[*i..j*] is given by *|S*[*i..j*]*| *= *j - i *+ 1. Furthermore, *S*[*i*..] denotes the *suffix *of *S *that starts at the *i*'th position, i.e., *S*[*i*..] = *S*[*i..N*]. Similarly, *S*[..*i*] denotes the *prefix *of *S *that ends at the *i*'th position, i.e., *S*[..*i*] = *S*[1..*i*].

### Chaos Game Representation (CGR) definition

The CGR iterative algorithm is constructed on a square in *IR*^2 ^where each of its vertex is assigned to a DNA symbol or base (*A, C, G, T*). For a given DNA sequence *S *of length *N*, CGR maps each prefix *S*[..*i*] onto a point *x_i _*∈ *IR*^2 ^following the iterative procedure:

(1) $$\left\{\begin{array}{c} x_{0}=\left(\frac{1}{2},\frac{1}{2}\right)\\ x_{i}=x_{i-1}+\frac{1}{2}\left(y_{i}-x_{i-1}\right),i=1,\ldots ,N \end{array}\right)\;\text{where }y_{i}=\left\{\begin{array}{c} \left(0,0\right)\text{ if }S\left[i\right]=A\\ \left(0,1\right)\text{ if }S\left[i\right]=C\\ \left(1,0\right)\text{ if }S\left[i\right]=G\\ \left(1,1\right)\text{ if }S\left[i\right]=T. \end{array}\right)$$

The *initial point x*_0 _in the original formulation [10] was taken as the square center, i.e., $x_{0}=\left(\frac{1}{2},\frac{1}{2}\right)$. Alternatively, this point could be chosen randomly on the square, *x*_0 _*~ *Unif([0, 1]^2^), or calculated as the last symbol *x*_0 _= *x_N _*by differently seeding the iterative function, providing symmetry adequate for circular genomes [23]. In addition, the *contraction ratio *of the original formulation is equal to $r=\frac{1}{2}$, which means that each iteration transforms the whole square into a sub-quadrant with length half the size. More generally, this value can be any number from $\frac{1}{2}\leq r<1$ to preserve bijectivity.

An alternative and non-recursive expression for *x_i _*as a function of all symbols *y*_1_,..., *y_i_*, contraction ratio *r*, and initial point *x*_0_, is given by

(2) $$\begin{array}{cccc} x_{i} & ={\left(1-r\right)}^{i}x_{0}+r\overset{i}{\underset{k=1}{\sum }}{\left(1-r\right)}^{k-1}y_{i-k+1} & \\ & ={\left(1-r\right)}^{i}x_{0}+r\overset{i}{\underset{k=1}{\sum }}{\left(1-r\right)}^{i-k}y_{k}, & & i=1,\ldots ,N. \end{array}$$

The generalization of CGR to higher-order alphabets was defined, in a more natural way, by extending the original CGR square to an hypercube [24], which can be bijectively mapped to 2D polygons by increasing the value of *r *[25]. The dimension *d *of the hypercube will depend on the length of the alphabet |Σ| as *d *= log_2 _|Σ| and all the remaining properties are maintained.

### CGR properties

One important property of CGR is the convergence of sequence coordinates in the space [0,1]^2 ^when the prefixes they represent share a common suffix. In fact, CGR maps prefixes with shared suffixes close to each other reflecting the property that, wherever the sequence context may be, the same suffix will be always mapped in the same region of CGR.

**Figure 1:**
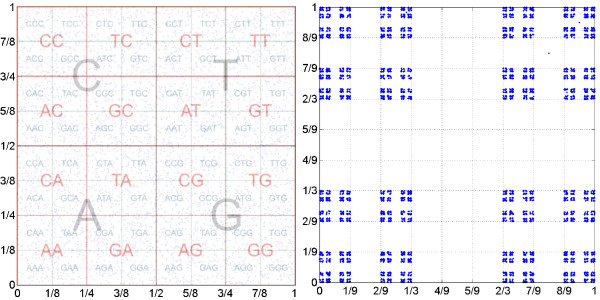
**CGR representation**. Chaos Game Representation (CGR) map of random DNA sequences of length *N *= 5,000. The first panel represents the original CGR proposal with contraction parameter $p=\frac{1}{2}$ and the superimposed sub-quadrants of the suffix labels. The second represents the same sequence but with $p=\frac{2}{3}$.

In the original CGR formulation where $r=\frac{1}{2}$, the distance between the coordinates is decreased by a factor of 2 in each common symbol in their suffixes. One consequence of this result is that the distance between the coordinates being less that 2*^-L ^*constitutes a necessary condition for sharing a *L*-long suffix. Another foregone conclusion is that CGR maps can be divided and labelled according to the corresponding substring, i.e., each substring is mapped onto a sub-square, creating a fractal-like structure. This allows the retrieval of Markov Chain transition probability tables [15], as exemplified graphically in Figure 1a. This result is not sensitive to the initial condition or point *x*_0 _chosen, since the Lyapunov exponent is negative.

More generally, in order to extract transition probability estimates for an *L*-order Markov chain, the interval [0,1] should be divided in 2^*L*+1 ^subintervals. The number of points in each sub-square thus created is then counted, which is the same as extracting the number of (*L*+1)-nucleotides. Extensions of this property allowed the estimation of *Variable Length Markov Models *(VLMM) [26,27] and time series forecasting by *Fractal Prediction Machines *(FPM) [28]. For other ratios *r *and initial points *x*_0 _these properties are all maintained. More recently, CGR further enabled a novel type of sequence analysis that associates its fractal geometry properties with biological structures [29].

### Constant-time longest common suffix via Cantor set embedding

As discussed previously CGR coordinates of prefixes sharing an *L*-long suffix are located within 2^-*L *^distance of each other. However, there are prefixes not sharing an *L*-long suffix that are also located within the same distance. We conclude that being in that neighborhood is not a sufficient condition to determine a common *L*-long suffix, as in some borderline cases the common suffix crudely taken from this fact is longer than the real one. In this section we address this shortcoming aiming at finding an efficient procedure to elicit longest common suffixes within CGR maps.

Intuitively, having not noticed that the aforementioned condition is only necessary, we would believe that the size of the longest common suffix of *S*_1_[..*i*] and *S*_2_[..*j*] is given by the greatest *ℓ *for which the *L*_∞_-norm

$${\left|x_{i}^{S_{1}}-x_{j}^{S_{2}}\right|}_{\infty }=\underset{b=1,2}{\text{max}}\left\{\left|x_{i:b}^{s_{1}}-x_{j:b}^{S_{2}}\right|\right\}$$

is still less than 2^-ℓ^, where *x_i _*= (*x*_*i*:1_, *x*_*i*:2_) for all *i*. Observe that the *L*_∞_-norm corresponds simply to the maximum distance on the two CGR dimensions. Finding such *ℓ *can be accomplished in constant time by the mean of a logarithm operation given by

(3) $$\left\lceil -{\text{log}}_{2}\left(|x_{i}^{S_{1}}-x_{j}^{S_{2}}{|}_{\infty }\right)\right\rceil -1.$$

To see why Eq. (3) fails to compute the longest common suffix, let *S*_1 _= *AT *and *S*_2 _= *TA*, therefore, *N *= 2. Moreover, *i *= *j *= 2. Clearly, the longest common suffix between *S*_1 _and *S*_2 _is 0, as they share no common suffix. However, using Eq. (3) we obtain

$$\left\lceil -{\text{log}}_{2}\left(|x_{2}^{S_{1}}-x_{2}^{S_{2}}{|}_{\infty }\right)\right\rceil -1=\left\lceil -{\text{log}}_{2}\left(|\left(0.625,0.625\right)-\left(0.375,0.375\right){|}_{\infty }\right)\right\rceil -1=\left\lceil -\text{log}\left(0.25\right)\right\rceil -1=1.$$

This indicates an incorrect common suffix of length *ℓ *= 1 between *S*_1 _and *S*_2_. If these strings are further extended to *S*_1 _= *A*(*A*)*T *and *S*_2 _= *T*(*T*)*A*, where (*α*) is a sequence of symbol *α *∈ Σ as long as wanted, then the corresponding CGR coordinates $x_{N}^{S_{1}}$ and $x_{N}^{S_{2}}$ will further approximate each other in the map and Eq. (3) will result in *ℓ *= *N *- 1, whereas their longest common suffix remains 0. We refer the intended reader to Additional file 1 where details on this analysis can be found.

The core of the problem lies on Eq. (3). Start by noticing that the logarithm in Eq. (3) corresponds to the number of leading zeros in the fractional part of $|x_{i}^{S_{1}}-x_{j}^{S_{2}}{|}_{\infty }$ when this *L*_∞_-norm is seen in binary numeral system (base 2). Intuitively, these leading zeros, say *L*, represent a match between *S*_1_[*i *- *L *+ 1...*i*] and *S*_2_[*j - L *+ 1...*j*]. The shortcoming of Eq. (3) is that the *L*_∞_-norm introduces an unexpected problem with the carry mechanism. To better see why, assume again that *S*_1 _= *AT *and *S*_2 _= *TA*, so *N *= 2. In this case we can write the CGR coordinates $x_{2}^{S_{1}}$ and $x_{2}^{S_{2}}$ in base 2 as $x_{2}^{S_{1}}=\left(0.\text{1}0\text{1},0.\text{1}0\text{1}\right)$ and $x_{2}^{S_{2}}=\left(0.0\text{11},0.0\text{11}\right)$ When computing the difference $x_{2}^{S_{1}}-x_{2}^{S_{2}}$ between the coordinates we get

$$\begin{array}{ccccc} & 0. & 1 & 0 & 1\\ - & 0. & 0_{1} & 1 & 1\\ & 0. & 0 & \;1 & 0 \end{array}\;\;,$$

and we come across a carry that masks the real dissimilarity between the last characters of string *S*_1 _and *S*_2 _(highlighted in bold). Indeed, the difference $x_{2}^{S_{1}}-x_{2}^{S_{2}}$ indicates that the first character of *S*_1 _and *S*_2 _match, which is not the case.

In order to solve the longest common suffix in constant time we must be able to compute the difference between CGR coordinates without incurring the penalty of the carry mechanism. We achieve this by embedding the CGR coordinates in the *Cantor set *[30,31]. For that, we use base 3, instead of base 2, admitting a numeral representation consisting entirely of 0's and 2's (never using the digit 1). A way to achieve this is to consider that usual CGR coordinates are expressed in base 3 by replacing all 1's with 2's. Such CGR coordinates can be computed similarly as in Eq. (2) by

(4) $$\begin{array}{cccc} x_{i} & =2\;\times \;3^{-i}\left(\frac{1}{3},\frac{1}{3}\right)+\overset{i}{\underset{k=1}{\sum }}2\times 3^{-k}y_{i-k+1} & \\ & =2\;\times \;3^{-i}\left(\frac{1}{3},\frac{1}{3}\right)+\overset{i}{\underset{k=1}{\sum }}2\;\times \;3^{k-i-1}y_{k}, & & i=0,\ldots ,N. \end{array}$$

In this case, the difference $x_{2}^{S_{1}}-x_{2}^{S_{2}}$ in the previous example becomes

$$\begin{array}{ccccc} & 0. & 2 & 0 & 2\\ - & 0. & 0_{1} & 2 & 2\\ & 0. & 1 & 1 & 0 \end{array}$$

indicating, as expected, that strings *S*_1 _and *S*_2 _mismatch in the first symbol.

This transformation generally leads to the desired scalability because the carry-on mechanism does not change the difference between the operands in the first bit where the difference takes place. This is a mandatory practice for the base-3 logarithm ${\text{log}}_{3}\left(|x_{2}^{S_{1}}-x_{2}^{S_{2}}{|}_{\infty }\right)$ correctly identifying the first bit where the difference between the coordinates is found. In the following, we show this fact in detail. Start by noticing that when dealing with the *L*_∞_-norm of the difference we only have to cope with differences where the left operand is greater than the right one. In that case, the dissimilarity may take place exactly in the first digit of the difference. If so, a carry-on may, or may not, take place just before moving to the second digit of the difference. Either way we end up with the same base-3 logarithm, as in both cases the first digit of the difference is greater than 0 (highlighted in bold), i.e.,

$$\begin{array}{ccccc} & 0. & 2 & \ldots & 2\\ - & 0. & 0 & \ldots & 2\\ & 0. & 2 & \ldots & 0 \end{array}\;\text{and}\;\begin{array}{ccccc} & 0. & 2 & \ldots & 2\\ - & 0. & 0_{1} & \ldots & 2\\ & 0. & 1 & \ldots & 0 \end{array}\;.$$

On the other hand, if the dissimilarity does not take place in the first digit of the difference, we conclude that the first bit of the difference greater than 0 will be exactly the first bit where the mismatch occurs, either with a carry-on or not. Indeed, assuming that the first *k *bits of the difference $x_{2}^{S_{1}}-x_{2}^{S_{2}}$ are similar we have that

$$\begin{array}{cccccccc} & 0. & b_{1} & \ldots & b_{k} & 2 & \ldots & 2\\ - & 0. & b_{1} & \ldots & b_{k} & 0 & \ldots & 2\\ & 0. & 0 & \ldots & 0 & 2 & \ldots & 0 \end{array}\;\text{and}\;\begin{array}{cccccccc} & 0. & b_{1} & \ldots & b_{k} & 2 & \ldots & 2\\ - & 0. & b_{1} & \ldots & b_{k} & 0_{1} & \ldots & 2\\ & 0. & 0 & \ldots & 0 & 1 & \ldots & 0 \end{array}\;\;,$$

correctly concluding that a mismatch occurs at the (*k *+ 1)'th bit of the difference and, therefore, *S*_1 _and *S*_2 _have a longest common suffix of size *k*.

In addition, we show that using CGR coordinates embedded in the Cantor set as in Eq. (4) we achieve a sufficient and necessary condition to compute longest common suffixes in constant time. For the sake of simplicity, and without loss of generality, we assume *N *= *M *and *i *= *j*.

**Theorem**. *Let S*_1 _*and S*_2 _*denote two strings of size N. We have that *${\left|x_{i}^{S_{1}}-x_{i}^{S_{2}}\right|}_{\infty }<3^{-L}$*if and only if S*_1_[*i - L *+ 1..*i*] = *S*_2_[*i - L *+ 1..*i*], *with *1 ≤ *L *≤ *i *≤ *N*.

**Proof: **We start by showing that if ${\left|x_{i}^{S_{1}}-x_{i}^{S_{2}}\right|}_{\infty }<3^{-L}$ then *S*_1_[..*i*] and *S*_2_[..*i*] have a common *L*-length suffix. This part of the proof follows by contradiction. Thus, assume that ${\left|x_{i}^{S_{1}}-x_{i}^{S_{2}}\right|}_{\infty }<3^{-L}$ and that *S*_1_[*i - L *+ 1..*i*] ≠ *S*_2_[*i - L *+ 1..*i*]. If *S*_1_[*i - L *+ 1..*i*] ≠ *S*_2_[*i - L *+ 1..*i*] then, in base 3 numeral representation,

we have that $x_{i:c}^{S_{d}}=0.b_{1}\ldots b_{k}2\ldots$ and $x_{i:c}^{S_{3-d}}=0.b_{1}\ldots b_{k}0\ldots ,$ for some 1 ≤ *k < L *and *c, d *= 1, 2. Then,

$$3^{-\left(k+1\right)}<x_{i:c}^{S_{d}}-x_{i:c}^{S_{3-d}}<2\;\times \;3^{-\left(k+1\right)}<3^{-k}.$$

This contradicts the hypothesis that ${\left|x_{i}^{S_{1}}-x_{i}^{S_{2}}\right|}_{\infty }<3^{-L}$, concluding this part of the proof.

We now proceed to prove that if *S*_1_[..*i*] and *S*_2_[..*i*] have a common *L*-length suffix then ${\left|x_{i}^{S_{1}}-x_{i}^{S_{2}}\right|}_{\infty }<3^{-L}$. Since by hypothesis *S*_1 _and *S*_2 _have a common *L*-length suffix we know that $y_{k}^{S_{1}}=y_{k}^{S_{2}}$ for all *i-L*+1 ≤ *k *≤ *i*. Hence, by Eq. (4) we have that

$$x_{i}^{S_{1}}-x_{i}^{S_{2}}=2\times \overset{i-L}{\underset{k=1}{\sum }}3^{k-i-1}\left(y_{k}^{S_{1}}-y_{k}^{S_{2}}\right).$$

The *L*_∞_-norm of this difference is then given by

$${\left|x_{i}^{S_{1}}-x_{i}^{S_{2}}\right|}_{\infty }={\left|2\times \overset{i-L}{\underset{k=1}{\sum }}3^{k-i-1}\left(y_{k}^{S_{1}}-y_{k}^{S_{2}}\right)\right|}_{\infty }\leq 2\times \left(3^{-L-1}+3^{-L-2}+\cdots +3^{-i}\right)<3^{-L}.$$

This concludes the proof.

Interestingly, this representation corresponds to a variant of CGR that uses a contraction ratio of $r=\frac{2}{3}$, instead of the original $r=\frac{1}{2}$, and an initial point in the set attractor $x_{0}=\frac{2}{3}$. In fact, this result is achieved by using a similar recursive expression as the one given by Eq. (1):

(5) $$\left\{\begin{array}{c} x_{0}=\left(\frac{2}{3},\frac{2}{3}\right)\\ x_{i}=x_{i-1}+\frac{2}{3}\left(y_{i}-x_{i-1}\right),\;i=1,\ldots ,N \end{array}\right)\;\;\;\text{where }y_{i}=\left\{\begin{array}{cc} \left(0,0\right) & \text{if }S\left[i\right]=A\\ \left(0,1\right) & \text{if }S\left[i\right]=C\\ \left(1,0\right) & \text{if }S\left[i\right]=G\\ \left(1,1\right) & \text{if }S\left[i\right]=T. \end{array}\right)$$

The graphical comparison between these two versions is given in Figure 1. The sub-quadrants in the first case are contiguous and, therefore, the closeness in this representation is a necessary but not a sufficient condition for sharing the same suffix. On the contrary, in the second representation, the sub-quadrants are separated by at least 3^-*L *^and so the condition becomes sufficient, as the border effect is absent for every *L*-long suffix.

## Results

In this section we present several exact and approximate string matching problems, along with their CGR solution. These include the longest common extension problem, as well as finding all palindrome and tandem repeats, exact and approximate string matching, and longest common substring. In addition, we show how CGR can be used as a rolling hash for the Rabin-Karp string search algorithm. A study of the time complexity attained by these algorithms is given throughout this section and a summary in provided in Table 1.

**Table 1:** Time complexity of different applications

Application	output size	standard CGR	quadtree CGR	Suffix Tree
LCE	1	*O*(1)	*O*(1)	*O*(1)
*k*-mismatch	*N*	*O*(*kN*)	*O*(*kN*)	*O*(*kN*)
Exact palindromes	*N*	*O*(*N*)	*O*(*N*)	*O*(*N*)
*k*-mismatch palindromes	*N*	*O*(*kN*)	*O*(*kN*)	*O*(*kN*)
Exact tandem repeats	*z*	*O*(*N *log *N *+ *z*)	*O*(*N *log *N *+ *z*)	*O*(*N *log *N *+ *z*)
*k*-mismatch tandem repeats	*z*	*O*(*kN *log *N *+ *z*)	*O*(*kN *log *N *+ *z*)	*O*(*kN *log *N *+ *z*)

Longest common suffix	1	*O*(1)	*O*(1)	*O*(1)
Exact match (MATCHES)	*occ*	*O*(*N*)	*O*(*L *+ *occ*)	*O*(*L *+ *occ*)
Occurrences (COUNT)	1	*O*(*N*)	*O*(*L *+ *occ*)	*O*(*L *+ *occ*)
Longest common substring	1	*O*(*N *log *N*)	*O*(*N *log *N*)	*O*(*N*)

This time complexity analysis does not include the pre-processing step of building the required indexes, as all depend solely in the size *N *of the input string *S*. In the following *L *is the length of pattern *P*.

All the code for both Mathematica^® ^and JavaScript implementations is made available at http://cgrsuffix.github.com/.

### CGR data structures and implementation

CGR was implemented using two different data structures, a standard floating-point matrix and a quadtree-like structure. In this section we describe these implementations along with the required notation.

#### Standard CGR index

The *standard implementation *uses a floating-point representation that calculates all the coordinates iteratively using Eq. (5). In this implementation, a string *S *of size *N *is represented in memory by a floating-point matrix of size *N × *2. This corresponds to a *N*-size array of pairs, denoted by $x^{S}=\left(x_{i}^{S},\;.\;.\;.\;,x_{N}^{S}\right)$, where the *i*'th entry $x_{i}^{S}=\left(x_{i:1}^{S},x_{i:2}^{S}\right)$ is accessed in *O*(1) time, for all 1 ≤ *i *≤ *N*.

This data structure is built in optimal time, that is, in time linear in the length of the string *S*. Indeed, it takes *O*(*N*) time to build the matrix and each entry of the matrix is computed in *O*(1) time. This constant time is achieved as, for all 1 ≤ *i *≤ *N*, coordinate $x_{i}^{S}$ can be computed from coordinate $x_{i-1}^{S}$ as in Eq. (5) by a fixed number of sum operations.

Depending on the application at hand we may need to build one or two standard CGR maps. These CGR maps may correspond to the direct DNA string as well as to its reverse. In addition, the complement or reverse complement may also be used. The *reverse *string of *S *is denoted by *S^r ^*and the *reverse complement *of *S *is denoted by *S^rc^*. If the string *S^rc ^*is reversed once again, the *complement *string *S^c ^*is obtained. Finally, given a string *S*, we call the CGR map *x^S ^*the *direct CGR index*, the CGR map $x^{S^{r}}$ the *reverse CGR index*, the CGR map $x^{S^{rc}}$ the *reverse-complement CGR index*, and the CGR map $x^{S^{c}}$ the *complement CGR index*.

#### Quadtree-based CGR index

Since in its simplest form a CGR map is just a set of coordinates on a two-dimensional plan, we can use a quadtree to index such points [32]. For this index, a tree data structure is used where each node has at most four children. Each child node represents a sub-quadrant of its parent node containing an extension of the parent prefix. Assuming Σ = {*A, C, G, T*}, we identify each child by *A, C, G *and *T *. Given a string *S *with size *N*, at most *N *nodes are stored in the quadtree as follows: for each prefix *S*[1..*i*] of *S*, we start inserting its reverse on the quadtree until we reach a leaf, where we insert a new node and stop. For instance, let *S *= *GACGA *and let us start with an empty quadtree [,,, ]. After inserting the prefix *G*, the quadtree becomes [,, 1: [,,,],] where the symbol *S*[1] is the unique symbol in quadrant *G*. After inserting prefixes *GA *and *GAC*, the quadtree becomes [2: [,,,], 3: [,,,], 1: [,,,],] as *S*[1], *S*[2] and *S*[3] are the unique points in quadrants *G, A *and *C*, respectively. But, after inserting the prefix *GACG*, the quadtree becomes [2: [,,,], 3: [,,,], 1: [, 4: [,,,],,],] as the quadrant *G *has a second point *S*[4] in the subquadrant *GC*. Similarly, after inserting the prefix *GACGA*, the quadtree becomes [2: [,, 5: [,,,],], 3: [,,,], 1: [, 4: [,,,],,],], where the quadrant *A *has a new second point in subquadrant *AG*.

Note that quadtrees as described are just *compressed tries *of *S *prefixes, also known as *CGR-trees*, described as quaternary trees related to *digital search trees *proposed by Cenac et al. [33]. Moreover, the use of a quadtree to index the CGR coordinates is equivalent to a suffix tree, up to an inversion of the text (because of the prefix/suffix duality). Hence, although as general search trees, quadtrees may degenerate and its construction becomes quadratic on string size, we can improve such construction to linear time in worst case using Ukkonen's algorithm [34] after an inversion of the text, as expected. For larger alphabets higher order trees, such as *octrees*, can be considered.

#### Web-based JavaScript implementation

Along with the Mathematica^® ^implementation, a JavaScript version is also provided, where an efficient binary representation of CGR coordinates is used, thus eliminating floating point operations. Additionally, this implementation also uses quadtrees for indexing CGR maps, improving search complexity by at least one order of magnitude when compared with traditional implementations.

We choose JavaScript as underlying language so that our implementation can be used in the Web distributed computational environment. The JavaScript interpreters, such as those found on modern browsers, include high performance features such as runtime compilation, achieving performances usually associated with scientific computing environments. Furthermore, it natively supports code migration (through the SCRIPT element of the browsers Document Object Model) which provides a seamless mechanism for code distribution and reuse.

### Longest common extension

Given two strings *S*_1 _and *S*_2_, each one with length *N *and *M*, and a long sequence of position pairs, the *longest common extension *(LCE) problem consists in finding, for a given query pair (*i, j*), the length of the longest prefix of *S*_1_[*i*..] that matches a prefix of *S*_2_[*j*..].

This problem is directly related with the longest common suffix described in the previous section, up to an inversion of the strings. Therefore, to address LCE queries with standard CGR indexes it suffices to search for shared suffixes in the reverse CRG indexes of *S*_1 _and *S*_2_, as that suffixes would represent prefixes in the direct indexes. The position pair (*i, j*) needs to be updated accordingly, to (*N *- *i *+ 1, *M *- *j *+ 1), in order to reference the correct positions in the reverse strings. With suffix/prefix duality problem solved we resort on CGR maps embedded in the Cantor set to perform LCE queries.

The pseudo-code to find the LCE of *S*_1_[*i*..] and *S*_2_[*j*..], with *|S*_1_*| *= *N *and *|S*_2_*| *= *M*, is given in Algorithm 1.

**Algorithm 1 **Longest common extension (LCE) of *S*_1 _and *S*_2 _for position pair (*i, j*)

LCE(standard CGR index entry $x_{N-i+1}^{{\text{S}}_{1}^{r}}$, standard CGR index entry $x_{M-j+1}^{{\text{S}}_{2}^{r}}$)

1. return $\left\lceil -{\text{log}}_{3}\left(|x_{N-i+1}^{{\text{S}}_{1}^{r}}-x_{M-j+1}^{{\text{S}}_{2}^{r}}{|}_{\infty }\right)\right\rceil -1$

This single step algorithm makes each LCE query answered in constant time. Many string algorithms use LCE as a subtask that needs to be addressed efficiently and will therefore benefit from this result, as shown in the following sections.

### Common operations on strings

In this section, several common operations on strings are analyzed and a comparison between CGR and traditional data structures is given when appropriate. This analysis focus on how each operation can be achieved through different CGR implementations, along with its respective time complexity.

Given a string *S *of size *N *and a pattern *P *of size *L*, searching for occurrences of *P *within *S *is probably the most common operation on strings. This problem, named MATCHES, has been utterly studied and there are well known algorithms to solve it in linear time on the length of *S *and *P*. The problem becomes more challenging if we are given a string *S *and we want to search, possible online, for a set of patterns within *S*. In this case, the preprocessing and indexing of *S *can greatly improve the search time as we just process *S *once, which is not the case if we use algorithms such as Knuth-Morris-Pratt [35] or Aho-Corasick [36]. In this context, the string *S *needs to be indexed and standard or quadtree-based CGR indexes can be used.

If a standard CGR index is used, the occurrences of *P *in *S *can be found by scanning *S *with LCE queries to match *P *in each suffix *S*[*i*..], with 1 ≤ *i *≤ *N - L *+ 1. More precisely, if the LCE query of *S *and *P *for a given position pair (*i*, 1) returns *L *then *P *matches *S*[*i*..*i *+ *L - *1]. The algorithm for computing all matches of a pattern *P *in a string *S *is given as Algorithm 2. Recall that we need to use the reserve CGR indexes of *S *and *P *to tackle suffix/prefix duality and all CGR coordinates are embedded in the Cantor set, thus, defined as in Eq. (5). Concerning time complexity, Step 2 takes *O*(*N *- *L*) and both Step 3 and Step 4 take *O*(1), hence, the overall complexity of MATCHES is *O*(*N*) as *L < < N*.

**Algorithm 2 **MATCHES with standard CGR index

MATCHES(standard CGR index $x^{S^{r}}$, pattern *P*)

1. let $x^{P^{r}}=\left(x_{1}^{P^{r}},\ldots ,x_{L}^{P^{r}}\right)$ be the CGR coordinates for *P^r^*

2. for (*i *from 1 to *N *- *L *+ 1)

3. let *k *= LCE $\left(x_{N-i+1}^{S^{r}},\;x_{L}^{P^{r}}\right)$//longest common prefix of *S*[*i*..] and *P *[1..]

4. if (*k *= *L*) then *P *occurs in *S*[*i*..*i *+ *L - *1]

Although standard CGR indexes can be space efficient, as we only need to store a coordinate for each text position, the MATCHES operation is not as efficient as with suffix tree indexes. Indeed, with suffix trees this operation takes only *O*(*L *+ *occ*) time, where *occ *is the number of occurrences of *P *in *S *(usually, *occ < < N*). Notwithstanding, we can improve the running time of MATCHES by using a quadtree-based CGR index, instead of a standard one. For this purpose, consider a quadtree for the CGR map of *S*. Given a pattern *P*, we take its reverse *P^r ^*and we descend on the quadtree until we either reach the end of *P^r ^*or a leaf of the quadtree. Along the descendent path we must check for each internal node if their CGR coordinates are equal to those of *P*, which takes constant time as in Algorithm 2. If this check is true, we must report an occurrence. If we reach the end of *P^r ^*and we are at node *n *with children, we must also check every node in the subtree rooted at *n *as each one matches the pattern. Hence, searching for a pattern *P *takes linear time on the size of *P *plus the number of its occurrences in *S*, i.e., *O*(*L *+ *occ*) as in suffix trees.

Another common operation on strings is COUNTS. It consists on determining the number of occurrences of a given pattern *P *in a string *S*. This can be easily achieved by adapting the MATCHES operation, for both standard and quadtree-based CGR indexes, resulting in the same time complexity. The only difference is that instead of outputting the occurrences a counter is used to retrieve the total number of occurrences of *P *in *S*.

Other common operations on strings include LONGEST COMMON SUFFIX, IS PREFIX and IS SUFFIX. The LONGEST COMMON SUFFIX was addressed in the Cantor set description, achieving constant time complexity as LCE. Given two strings *S *and *P *, with *|S| *= *N, |P| *= *N *and *L < N*, the IS SUFFIX operation consists in checking if *P *appears as a suffix of *S*. This can be done by computing the LCE for coordinates $x_{L}^{P}$ and $x_{N}^{S}$. The answer to IS SUFFIX is true if and only if the LCE query returns *L*, otherwise the answer is false. The IS PREFIX operation consists in checking if *P *appears as prefix of *S *and it can be easily done by reversing both *S *and *P *and applying the previous operation. Since both IS SUFFIX and IS PREFIX operations rely solely in LCE, they achieve constant time complexity as long as the needed CGR indexes are already computed.

We conclude this section by discussing four new operations that are specific to CGR but related with relevant operations in suffix trees (namely, SLINK and WEINERLINK). As we shall see later these operations provide a way for performing incremental hashing, an approach used for Rabin-Karp string matching algorithm. The LEFT DELETION of a string *S *of size *N *is the substring of *S *from which the first letter was taken, that is, given *S *= *αP*, the LEFT DELETION of *S *is *P*. Given the last CGR coordinate of *S*, denoted by $x_{N}^{S}$, the new CGR coordinate corresponding to the LEFT DELETION of *S *is given by

(6) $$x_{N-1}^{P}=x_{N}^{S}-r{\left(1-r\right)}^{N-1}\left(y_{1}^{S}-x_{0}\right).$$

Moreover, the LEFT INSERTION compares what happen when we add a symbol before the string, that is, given a string *S *the LEFT INSERTION of *α *in *S *is *Q *= *αS*. Given the last CGR coordinate of *S*, denoted by $x_{N}^{S}$, the new CGR coordinate corresponding to the LEFT INSERTION of *α *in *S *becomes

$$x_{N+1}^{Q}=x_{N}^{S}+r{\left(1-r\right)}^{N+1}\left(y_{1}^{Q}-x_{0}\right).$$

Clearly, this operations are computed in constant time. Finally, we recall that symmetric operations of RIGHT INSERTION and RIGHT DELETION can be straightforwardly computed from the iterative definition of CGR in Eq. (1) also with *O*(1) time complexity.

**Figure 2:**
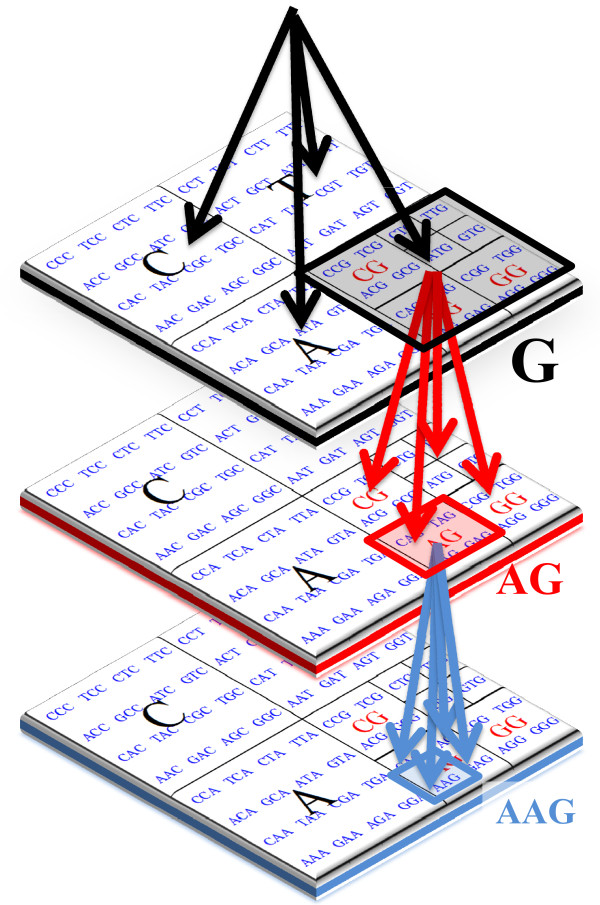
**CGR and Suffix Tree comparison**. CGR and suffix tree comparison. The CGR of each suffix is close in the map, which relates directly to suffix trees structure.

The examples presented in this section illustrate the algorithmic equivalence between CGR and suffix tress. Graphically, this can be observed through the representation of both data structures, given in Figure 2.

### Finding palindromes and tandem repeats

This section presents two applications of CGR based on exact string matching that rely on LCE described above. A nucleotide sequence *S *is said to be a *(complemented) palindrome *if it is equal to its reverse complement. In this case, the first half of *S *converted to its reverse complement is equal to the second half of *S*. If the two halves of the palindrome are not adjacent it is said to be a *separated palindrome*. Moreover, the palindrome is said to have a *radius *correspondent to the size of each half. Palindromic sequences play an important role in molecular biology. They are, for instance, specifically recognized by many restriction endonucleases [37] and usually associated with methylation sites [38].

The problem of finding all palindromes focus on uncovering all maximal complemented palindromes from a nucleotide sequence. By maximal it means that only the bigger palindrome is extracted. For instance, the sequence *TTATAA *has a palindrome of radius 1 (*AT*), a palindrome of radius 2 (*TATA*), and a palindrome of radius 3 (*TTATAA*), but only the latter is maximal.

The algorithm to find all palindromes uses, as subtask, forward and backward LCE queries from the midpositions of potential palindromes. The backward query corresponds, actually, to a forward query over the reverse complement of *S*. The detailed pseudo-code of this algorithm can be found in Addition file 2. Since each of the aforementioned extensions can be performed over a reverse and a complement CGR indexes in constant time, the algorithm takes *O*(*N*) time, corresponding to the time needed to cover all string *S*.

In practice, palindromes are often separated over the DNA sequence. If the gap between the two halves of the palindrome is fixed, it happens to be a simple variant of the previous problem. Therein, forward and backward extensions are performed in positions apart of each other (Additional file 2), a procedure that is again solved in linear time. The general case of finding all separated palindromes, without a fixed gap, is more complex as it is equivalent to the problem of finding all inverted repeats. As expected, an *inverted repeat *is a sequence of nucleotides that is the reverse complement of another sequence that is found further downstream the DNA sequence. These sequences are usually transposon boundaries [39,40].

Another major application that benefits from constant-time LCE queries is finding all tandem repeats in a nucleotide sequence. *Tandem repeats *are adjacent copies of a same DNA subsequence. They usually consist of short copies of 2-6 base pairs in length repeated throughout all eukaryotic genomes. Tandem repeats often represent important control sequences, such as upstream promoter sequences [41]. In addition, they have been used in forensic science for determining parentage [42].

The problem entailing tandem repeats focus in locating all tandem repeats in a nucleotide sequence. For instance, the sequence *TTATTA *has a tandem repeat *TT *of radius 1 and a tandem repeat *TTATTA *of radius 3, both starting in the first position of the nucleotide sequence. It also contains a tandem repeat *TT *of radius 1 at position 4.

The algorithm to find for all tandem repeats was first proposed by Landau and Schmidt [43]. It is a divide and conquer algorithm that recursively break down the problem of finding tandem repeats into sub-problems, until these become simple enough to be solved directly. These simpler problems can be solved through two LCE queries to elicit the presence of a tandem repeat. The time complexity of the algorithm is *O*(*N *log *N *+ *z*) where *z *is the number of tandem repeats in *S*. The details of the algorithm, which requires the direct and reverse CRG indexes to be built in a pre-processing phase, are presented in Additional file 2.

### Approximate string matching

The *k-mismatch *problem is an approximate matching problem where *k *mismatches are allowed. Approximate matching is a mandatory task in molecular biology as DNA sequence patterns are usually described in an approximate way. This makes way for more relaxed versions of palindrome and other repeat problems. Landau and Vishkin [44] and Myers [45] were the first to present an *O*(*kN*) solution for the more general *k*-differences problems, where insertions and deletions are also considered.

The idea of the algorithm for approximate matching stems from the fact that extensions may be intercalated with mismatches. Therefore, if *k *mismatches are allowed, *k *+ 1 extensions may be performed to match a pattern *P *of size *L *in a substring *S*[*i*..*i *+ *L - *1], with 1 ≤ *i *≤ *N - L *+ 1. Roughly speaking, match operations are as follows: (i) perform a LCE query to match *P*[1..*L*] in *S*[*i*..*i *+ *L-*1]; (ii) if a mismatch takes place in position *j < L *of *P*, jump over the mismatch and go to (i) to perform another LCE query to match *P*[*j *+ 1..*L*] in *S*[*i *+ *j *+ 1..*i *+ *L *+ 1]. This procedure must be undertaken recursively until *k *mismatches are reached or the end of *P *is reached. If the extensions does not reach the end of *P *then more than *k *mismatches are needed. Conversely, if the extensions reach the end of *P *then *P*[1..*L*] matches *S*[*i*..*i *+ *L - *1] with at most *k *mismatches.

Concerning time complexity, each match operation takes at most *O*(*k*) time, since at most *k *+ 1 extensions are tried and each extension takes *O*(1). The scan over all string *S *takes *O*(*N*), therefore the *k*-mismatch problem takes *O*(*Nk*) time. A detailed explanation, as well as the pseudo-code of the algorithm, can be found in Additional file 2. This procedure needs only the reverse CGR index of *S*, without using any special index data structure.

A simple variant of the *k*-mismatch algorithm can be used to solve the all *k*-mismatch palindromes. The idea is always to perform an extension and jump over a mismatch to perform another extension until the number of allowed mismatches are attained. Consequently, the complexity of all *k*-mismatch palindromes is updated to *O*(*Nk*) time. The algorithm for all *k*-mismatch tandem repeats [46] is also coincident, although more complex, and is fully described in the Additional file 2.

### Longest common substring

The retrieval of the longest common substring of two strings is a classical computer science problem. Given a string *S*_1 _of size *N *and a string *S*_2 _of size *M*, a naive approach to this problem simply consider all positions *i *in *S*_1 _and *j *in *S*_2 _and proceed in both strings by matching symbols, until a mismatch is found. Such procedure takes *O*(*NM*^2^) time. By using linear time search algorithms, such as the Knuth-Morris-Pratt, this result can be improved to *O*(*NM*). Still, the performance of these naive solutions pales in comparison with the linear time performance of suffix trees [4].

In the CGR framework we propose an algorithm that does not achieve linear time complexity, as suffix tree does, but it is sufficiently close to warrant a careful analysis. We start this analysis by considering the simpler problem of finding duplicates in a list of numbers. Assume we are given an unordered list of numbers *ℓ*, of size *N*, and we wish to determine if there are any repeated number. A naive solution would compare every pair of numbers to check if they are equal, requiring *O*(*N*^2^) time. A more sophisticated solution would be to sort the list *ℓ *and check only consecutive numbers. This algorithm requires overall *O*(*N *log *N*) time, where the sorting step is the bottleneck of the procedure.

Our solution for determining the longest common substring with CGR is similar to this procedure. We sort the CGR coordinates according to the quadrant they appear on. First, the ones close to (0, 0), then the ones close to (0, 1), then the ones close to (1, 1), and finally the ones close to (1, 0). If two points are in the same quadrant then they are sorted according to the second quadrant that they appear on, or iteratively by the first quadrant that they differ. The advantage of having LCE queries in constant time within CGR maps is that two CGR coordinates can be efficiently compared. First, we use LCE to skip the quadrants that the two coordinates share and, then we use the resulting value to order the two. Hence, it is possible to sort all the CGR coordinates in *O*(*N *log *N*) time.

To obtain the longest common substring from the CGR representation we join the CGR coordinates from *S*_1 _and *S*_2 _and sort them all together, according to the previous procedure. Then, we traverse the sorted list and, if two consecutive coordinates come from two different strings, i.e., one from *S*_1 _and the other from *S*_2_, we compute the LCE between the two. Overall our goal is to determine the pair that has the largest LCE value, as this corresponds the longest common substring among the two strings.

### Rabin-Karp string matching algorithm

The procedure for calculating the CGR coordinates (Eq. (1) and (2)) bare a striking similarity to Rabin-Karp string matching algorithm [47]. This algorithm uses a *rolling hash function h*, from strings to integers, allowing to reduce the problem of comparing strings to the problem of comparing integers, which can be computed much more efficiently in commodity hardware.

Given a string *S *of size *N *and a pattern *P *of size *L*, the Rabin-Karp algorithm starts by computing the code *h*(*P*). Then, all *L*-tuples of *S *are scanned in order to check if *h*(*S*[*i*..*i *+ *L *- 1]) = *h*(*P*), with 1 ≤ *i *≤ *N - L *+ 1. The substrings for which *h*(*P*) = *h*(*S*[*i*..*i *+ *L - *1]) are potential occurrences of *P*, which should be further checked naively. This algorithm was one of the firsts to achieve linear pattern matching efficiency, taking only *O*(*N*) time to scan a string of size *N*. Notwithstanding, it results in a randomized algorithm, since *h*(*P*) = *h*(*S*[*i*..*i *+ *L - *1]) does not constitute a guarantee that *P *= *S*[*i*..*i *+ *L - *1] and so, an additional check is needed to verify if *P *= *S*[*i*..*i *+ *L - *1]. In consequence, its performance is only *O*(*N*) in the expected case, and the worst case may be significantly worse, taking *O*(*N*^2^) time. Other linear time algorithms such as the Knuth-Morris-Pratt and the Boyer-Moore can guarantee linear time performance, even in the worst case [35,48].

The Rabin-Karp algorithm has the advantage of producing and index as a side effect, which is actually a hash. Indexes have the advantage that they support subsequent string searches in much less time than searching over the all string *S*. However, their performance depend largely on the nature of the underlying hash. If the hash values of consecutive substrings are computed in constant time, it is possible to achieve almost a *O*(*L *+ *occ*) search time, meaning that searching for all the occurrences *occ *of a *L*-long pattern *P *can be performed in expected optimal time [49]. This is exactly what is attained when CGR is used to induce a rolling hash function for the Rabin-Karp procedure.

In the context of CGR, a possible rolling hash function can be constructed iteratively based on the CGR definition (Eq. (1)) and the LEFT DELETION operation described in Eq. (6). If one uses directly the coordinates of the last symbol of the first *L*-tuple to compute the first hash value we have that

$$h\left(S\left[1..L\right]\right)=x_{L}^{S}={\left(1-r\right)}^{L}x_{0}+r\overset{L}{\underset{k=1}{\sum }}{\left(1-r\right)}^{L-k}y_{k}^{S}.$$

The next hash value *h*(*S*[2..*L*+1]) can be again defined as the last CGR coordinate of string *S*[2..*L*+1]. This coordinate $x_{L}^{S\left[2..L+1\right]}$ can be computed from $x_{L}^{S\left[1..L\right]}=x_{L}^{S}$ by performing a LEFT DELETION and applying a new CGR iteration. The computation of this value is straightforward. First, the coordinate $x_{L-1}^{S\left[2..L\right]}$ is obtained by canceling symbol *S*[1] from $x_{L}^{S}$, i.e.,

$$x_{L-1}^{S\left[2..L\right]}=x_{L}^{S}-r{\left(1-r\right)}^{L-1}\left(y_{1}^{S}-x_{0}\right).$$

Then, to obtain the intended coordinate $x_{L}^{S\left[2..L+1\right]},$ a CGR iteration is applied and we get

$$x_{L}^{S\left[2..L+1\right]}=\left(1-r\right)x_{L-1}^{S\left[2..L\right]}+ry_{L+1}^{S},$$

which can be defined in terms of $x_{L}^{S}$ as

$$x_{L}^{S\left[2..L+1\right]}=\left(1-r\right)x_{L}^{S}-r{\left(1-r\right)}^{L}\left(y_{1}^{S}-x_{0}\right)+ry_{L+1}^{S}.$$

Thus, the Rabin-Karp CGR-based procedure starts by defining $h\left(S\left[\text{1}..L\right]\right)=x_{L}^{S}$ and proceeds by computing iteratively the code for *h*(*S*[*i *+ 1..*i *+ *L*]) as

(7) $$h\left(S\left[i+1..i+L\right]\right)=\left(1-r\right)\;\cdot \;h\left(S\left[i..i+L-1\right]\right)-r{\left(1-r\right)}^{L}\left(S\left[i\right]-x_{0}\right)+r\;\cdot \;S\left[i+L\right],\;i=1,\ldots ,N-L.$$

Even though this expression uses vectors and is therefore slightly more sophisticated than the most common expressions, CGR function is essentially a hash, except for the fact the we are not using remainders as in the original proposal. However, due to the limitation of the floating point representation, iterating Eq. (1) will eventually overflow, hence producing the same effect as explicitly computing the remainders. If this occurs for *h*(*P*) a spurious hit might be obtained, requiring a naive verification. Naturally, infinite precision techniques can be used to prevent this phenomena, which in turn yield powerful computation models.

Finally, with Eq. (7) it is possible to update the hash value in constant time and, therefore, obtaining all the hash values takes only *O*(*N*) for a string *S *of size *N*. Likewise a hash data structure that indexes all *L*-tuples of *S *can be built within this time.

## Conclusions

Graph-based data structures such as suffix trees are heavily applied in sequence comparison and bioinformatics problems, having attained a high performance level. This optimization makes them the most common data structures for string-related operations.

CGR has been, for more than two decades, a fruitful methodology for biological sequence comparison, providing a support for aligment-free analysis along with conveying an appealing pattern visualization capacity for whole genomes. In this work we have shown that CGR can go beyond these applications and demonstrate how typical string operations can be recoded and solved using CGR. We further illustrate the similarities between these two data structures for biological sequence analysis, showing that numerical biosequence representations given by chaos game iterative function systems constitute an alternative and competitive approach for common string-matching problems.

The applications presented in this work have focused on the longest common extension problem that, after linear time preprocessing of a standard CGR index, it is shown to be answered in constant time. This result allows to efficiently solve other string matching problems such as searching for palindromes, tandem repeats and matches with mismatches. Additionally, it is shown that CGR can be used as an hash function and its relation with Rarbin-Karp algorithm is highlighted.

The chief advantage of CGR is its simplicity and easy implementation. In addition, a more complex placement of the CGR coordinates in memory actually provides an efficient way to solve more demanding problems, for example, through the use of quadtrees, here proposed to solve the longest common substring problem.

The choice between numerical or graphical resolution is ultimately decided by the efficiency of the implementation. To assist in this selection we provide a summary of time complexities achieve by both data structures for a set of pertinent problems in string processing for molecular biology, showing the CGR parallelism with suffix trees in some of the problems addressed.

The operations analyzed covered typical string problems without being exhaustive. Possible extensions to be explored numerically include sequence alignment, which can be implemented using a dynamic programming approach in a matrix of all the pairwise distances between the CGR coordinates. The longest common subsequence can also be addressed in the future using numerical reasoning.

The comparison of CGR and suffix trees algorithms for biological sequence analysis provides a useful bridge between graph and numerical-based formalisms such that solutions to difficult problems can make use of both representations.

## Competing interests

The authors declare that they have no competing interests.

## Authors' contributions

SV conceived the study and contributed for the design of the experiments. AMC aimed the research to achieve longest common extension in constant time with standard CGR indexes. APF implemented the JavaScript version and analyzed the different implementations. LR analyzed the CGR against string processing algorithm such as suffix trees and Rabin-Karp. JSA identified programming environment and contributed to the discussion of results. All authors contributed to the writing of the manuscript. All authors have read and approved the final manuscript.

## Supplementary Material

Additional file 1 **Longest common extension queries**. Additional file 1 presents in detail the problem of computing LCE queries with CGR maps that uses a contraction parameter ratio of $r=\frac{1}{2}$. It also presents the solution by embedding the CGR coordinates in the Cantor set and shows that this corresponds to the CGR map with $r=\frac{2}{3}$. Click here for file

Additional file 2 **Detailed algorithms**. Additional file 2 presents in detail the algorithms sketched throughout the section that exploits LCE queries in constant time. Click here for file
