# Supplementary material for: Pattern matching through Chaos Game Representation: bridging numerical and discrete data structures for biological sequence analysis
Source: Algorithms Mol Biol. 2012 May 2;7:10. doi: 10.1186/1748-7188-7-10 (PMC3402988; doi:10.1186/1748-7188-7-10)
Supplement: Additional file 1 — Longest common extension queries. Additional file 1 presents in detail the problem of computing LCE queries with CGR maps that uses a contraction parameter ratio of r=12. It also presents the solution by embedding the CGR coordinates in the Cantor set and shows that this corresponds to the CGR map with r=23. [file 1748-7188-7-10-S1.PDF]

Additional file 1

“Pattern matching through Chaos Game Representation: Bridging numerical and discrete data structures for biological sequence analysis”

Susana Vinga, Alexandra M. Carvalho, Alexandre P. Francisco, Luís M. S. Russo and Jonas S. Almeida

LCE queries in constant time

This additional file details the computation of longest common extension (LCE) using standard CGR indexes. As described in the main text constant-time LCE queries require embedding CGR coordinates in the Cantor set.

We start by introducing CGR maps and some auxiliar functions. Next, we illustrate the problem of crudely using the distance between the CGR coordinates to compute longest common suffixes. We show that this criterion constitutes a necessary but not a sufficient condition, therefore leading to an optimistic result for the longest common suffix. We then reformulate the problem of finding longest common suffixes into longest common prefixes, and present the constant-time algorithm for finding LCE. We end up showing that embedding CGR coordinates in the Cantor set is equivalent to use a contraction ratio of r=2/3, instead of the commonly used r=1/2, with an initial point in the set attractor of x0=2/3.

All the examples given will be acompained with code in *Mathematica*. This *Mathematica* notebook is available at http://cgrsuffix.github.com/.

CGR map

We start by coding in *Mathematica* the plain CGR coordinates, for a given DNA sequence, as given by Eq. (2).

```
x0 = {0.5, 0.5};

x = Function[{i, y}, Power[2, -i] x0 + Sum[Power[2, -k] y[[i - k + 1]], {k, 1, i}]];
```

The previous definition of x is also equivalent to xx:

```
xx = Function[{i, y}, Power[2, -i] x0 + Sum[Power[2, k - i - 1] y[[k]], {k, 1, i}]];
```

We now set out to introduce some auxilar functions. The reverse function is used to reverse a DNA sequence.

```
reverse = Function[{S}, Module[{chars, l, i, temp},
    chars = Characters[S];
    l = Length[chars];
    i = 1;
    While[i <= Floor[l / 2],
     temp = chars[[i]];
     chars[[i]] = chars[[l - i + 1]];
     chars[[l - i + 1]] = temp;
     i++;
    ];
    StringJoin[chars]
   ]];

reverse["AAATTCG"]

GCTTAAA
```

The translateCintoCoordinate function just translate a single symbol over the DNA alphabet into the corresponding CGR coordinate.

```
translateCintoCoordinate = Function[{C},
   If[C == "A", {0, 0},
    If[C == "C", {0, 1},
     If[C == "G", {1, 0},
      If[C == "T", {1, 1}]]]]
  ];

Map[translateCintoCoordinate, Characters["ACGTTGCA"]]

{{0, 0}, {0, 1}, {1, 0}, {1, 1}, {1, 1}, {1, 0}, {0, 1}, {0, 0}}
```

Computing longest common suffixes within CGR maps

In this section we intend to illustrate that using the distance between the coordinates to elicit common suffixes results in an optimistc criterion. The longest common suffix as discussed in Eq. (3) of the manuscript is as follows:

```
LongestCommonSuffix =
  Function[{S1, S2, i, j}, Module[{yS1, yS2, N1, N2, xS1, xS2},
    (*reverse and translate strings into CGR*)
    yS1 = Map[translateCintoCoordinate, Characters[S1]];
    yS2 = Map[translateCintoCoordinate, Characters[S2]];
    (*compute their lengths*)
    N1 = Length[yS1];
    N2 = Length[yS2];
    (*compute standard CGR index entries*)
    xS1 = x[i, yS1];
    xS2 = x[j, yS2];
    (*LongestCommonSuffix result*)
    Ceiling[-Log[2, Max[Abs[xS1 - xS2]]]] - 1
   ]];
```

Two examples without boundary problems

Computing LongestCommonSuffix("GATTCC","GGTTCC",6,6) should give 4, as both strings share the suffix "TTCC" ending in the given position pair (6,6). The result that is given by the LongestCommonSuf-

fix function is the one that is expected. The example follows:

```
S1 = "GATTCC"; S2 = "GGTTCC"; LongestCommonSuffix[S1, S2, 6, 6]
```

```
4
```

Other example gives also the correct longest common suffix answer:

```
S1 = "AAAAT"; S2 = "TTTTA"; LongestCommonSuffix[S1, S2, 5, 5]
```

```
0
```

Two examples with boundary problems

Computing LongestCoomonSuffix("TTTTTTA","AAAAAAT",7,7) should give 0, as strings do not share a single suffix ending in the given position pair (7,7). However, the result that is given by the previous LongestCommonSuffix function is 6, which is incorrect. The example follows:

```
S1 = "TTTTTTA"; S2 = "AAAAAAT"; LongestCommonSuffix[S1, S2, 7, 7]
```

```
6
```

A simpler example that gives also the incorrect longest common suffix answer:

```
S1 = "TA"; S2 = "AT"; LongestCommonSuffix[S1, S2, 2, 2]
```

```
1
```

Computing longest common prefixes within CGR maps

Herein, we just translate the problem of finding longest common suffixes into longest common prefixes. The boundary problems will be adressed only in the next section.

```
LongestCommonPrefix =
  Function[{S1, S2, i, j}, Module[{yS1, yS2, N1, N2, xS1, xS2},
    (*reverse and translate strings into CGR*)
    yS1 = Map[translateCintoCoordinate, Characters[reverse[S1]]];
    yS2 = Map[translateCintoCoordinate, Characters[reverse[S2]]];
    (*compute their lengths*)
    N1 = Length[yS1];
    N2 = Length[yS2];
    (*compute standard CGR index entries*)
    xS1 = x[N1 - i + 1, yS1];
    xS2 = x[N2 - j + 1, yS2];
    (*LongestCommonPrefix result*)
    Ceiling[-Log[2, Max[Abs[xS1 - xS2]]]] - 1
   ]];
```

Two examples without boundary problems

Computing LongestCommonPrefix("GGTTTT","GGTTCC",1,1) should give 4, as both string share the prefix "GGTT" starting in the given position pair (1,1). The result that is given by the previous LongestCommonPrefix function is the one that is expected. The example follows:

```
S1 = "GGTTTT"; S2 = "GGTTCC"; LongestCommonPrefix[S1, S2, 1, 1]
```

```
4
```

Other example gives also the correct LongestCommonPrefix answer:

```
S1 = "AAAAT"; S2 = "TTTTA"; LongestCommonPrefix[S1, S2, 1, 1]
```

```
0
```

■ Two examples with boundary problems

Computing LongestCommonPrefix("TAAAAA","ATTTTT",1,1) should give 0, as strings do not share a single prefix starting in the given position pair (1,1). However, the result that is given by the previous LongestCommonPrefix function is 5, which is incorrect. The example follows:

```
S1 = "TAAAAA"; S2 = "ATTTTT"; LongestCommonPrefix[S1, S2, 1, 1]
```

```
5
```

A simpler, although similar, example has the same problem:

```
S1 = "TA"; S2 = "AT"; LongestCommonPrefix[S1, S2, 1, 1]
```

```
1
```

Constant-time LCE queries

■ Embedding CGR coordinates in the Cantor set

In the Cantor set the CGR coordinates are given by Eq. (4) as :

```
x0C = {1 / 3, 1 / 3};
```

```
xxC = Function[{i, y},
   2 * Power[3, -i] x0C + Sum[2 * Power[3, k - i - 1] y[[k]], {k, 1, i}]];
```

■ Computing LCE

The LCE function is similar to the previous LongestCommonPrefix function but now uses the CGR coordinates embedded in the Cantor set, as in Eq. (4), and a base-3 logarithm (instead of a base-2 logarithm):

```
LCE = Function[{S1, S2, i, j}, Module[{yS1, yS2, N1, N2, xS1, xS2},
    (*reverse and translate strings into CGR*)
    yS1 = Map[translateCintoCoordinate, Characters[reverse[S1]]];
    yS2 = Map[translateCintoCoordinate, Characters[reverse[S2]]];
    (*compute their lengths*)
    N1 = Length[yS1];
    N2 = Length[yS2];
    (*compute CGR index entries embedded in Cantor set*)
    xS1 = xxC[N1 - i + 1, yS1];
    xS2 = xxC[N2 - j + 1, yS2];
    (*LCE result*)
    Ceiling[-Log[3, Max[Abs[xS1 - xS2]]]] - 1
   ]];
```

■ Two plain examples

Computing LCE("GGTTTT","GGTTTT",1,1) should give 4, as both strings share a common prefix "GGTT". The example follows:

```
S1 = "GGTTTT"; S2 = "GGTTCC"; LCE[S1, S2, 1, 1]
```

```
4
```

Other plain example follows:

```
S1 = "GGTTCT"; S2 = "GGTTCC"; LCE[S1, S2, 1, 1]
```

```
5
```

- **The same two examples with boundary problems when computing LongestCommonPrefix with CGR coordinates given by Eq. (1)**

Computing LCE("AAAAT","TTTTA",1,1) should give 0, as both strings share no common prefix. We have seen before that when CGR coordiantes are given as in Eq. (1) a boundary problems shows up. However, when embedding CGR coordinates in the Cantor set we get the expected result:

```
S1 = "TAAAAA"; S2 = "ATTTTT"; LCE[S1, S2, 1, 1]
```

```
0
```

A simpler, although similar, example achieve the same result:

```
S1 = "TA"; S2 = "AT"; LCE[S1, S2, 1, 1]
```

```
0
```

Using a contraction parameter ratio of 2/3

To illustrate that embedding CGR coordinates in the Cantor set is equivalent to use a contraction ratio of r=2/3, instead of r=1/2 as usual, we show that Eq. (5) has as solution Eq. (4):

```
Eq4 = 2 * 3^(-i) * (1 / 3) + Sum[2 * 3^(k - i) y[k + 1], {k, 0, i - 1}]
```

$$2 \times 3^{-1-i} + \sum_{k=0}^{-1+i} 2 \times 3^{-i+k}\, y[1+k]$$

```
Clear[x];
RSolve[{x[0] == 2 / 3, x[i] == x[i - 1] + 2 / 3 (y[i] - x[i - 1])}, x[i], i]
```

$$\left\{\left\{x[i] \to 3^{-1-i}\left(2 + 9 \sum_{K[1]=0}^{-1+i} 2 \times 3^{-1+K[1]}\, y[1+K[1]]\right)\right\}\right\}$$

$$\textbf{Eq5 = Simplify}\left[3\wedge(-1-i)\left(2 + 9 \sum_{k=0}^{-1+i} 2 \times 3^{-1+k}\, y[1+k]\right)\right]$$

$$3^{-1-i}\left(2 + 9 \sum_{k=0}^{-1+i} 2 \times 3^{-1+k}\, y[1+k]\right)$$

```
Simplify[Eq4 - Eq5]
```

$$-3^{1-i} \sum_{k=0}^{-1+i} 2 \times 3^{-1+k}\, y[1+k] + \sum_{k=0}^{-1+i} 2 \times 3^{-i+k}\, y[1+k]$$

```
3^(1 - i) * 3^(-1 + k) == 3^(-i + k)
```

```
True
```
