# Supplementary material for: Pattern matching through Chaos Game Representation: bridging numerical and discrete data structures for biological sequence analysis
Source: Algorithms Mol Biol. 2012 May 2;7:10. doi: 10.1186/1748-7188-7-10 (PMC3402988; doi:10.1186/1748-7188-7-10)
Supplement: Additional file 2 — Detailed algorithms. Additional file 2 presents in detail the algorithms sketched throughout the section that exploits LCE queries in constant time. [file 1748-7188-7-10-S2.PDF]

Additional File 2

"Pattern matching through Chaos Game Representation: Bridging numerical and discrete data structures for biological sequence analysis"

Susana Vinga   Alexandra M. Carvalho   Alexandre P. Francisco
Luís M. S. Russo   Jonas S. Almeida

Contents

1 Introduction **2**

2 Notation **2**

3 Exact string matching **2**

3.1 Finding all palindromes 2

3.2 Finding all tandem repeats 4

4 Approximate string matching **6**

4.1 Finding all k-mismatches of a pattern 6

4.2 Finding all k-mismatch palindromes 7

4.3 Finding all k-mismatch tandem repeats 7

1 Introduction

This additional file presents detailed algorithms sketched throughout the main text.

We start by introducing notation, and then describe exact and approximate string matching algorithms that take advantage of constant-time LCE queries computed from standard CGR indexes.

2 Notation

Let S denote a string of size N. In all what follows, $S[i]$ represents the i'th symbol of S, with $1 \leq i \leq N$. Each $S[i]$ is a DNA symbol representing each nucleotide or base (A, C, G, T), that is, $S[i] \in \Sigma = \{A, C, G, T\}$. Moreover, $S[i..j]$ is the *substring* of S that starts at the i'th and ends at the j'th positions of S, with $1 \leq i \leq j \leq N$. The *length* of substring $S[i..j]$ is given by $|S[i..j]| = j - i + 1$. Furthermore, $S[i..]$ denotes the *suffix* of S that starts at the i'th position, i.e., $S[i..] = S[i..N]$. Similarly, $S[..i]$ denotes the *prefix* of S that ends at the i'th position, i.e., $S[..i] = S[1..i]$.

In addition, we introduce a few additional notations that apply to CGR maps. The *reverse* string of S is denoted by S^r and the *reverse complement* of S is denoted by S^{rc}. If the string S^{rc} is reversed once again, the *complement* string S^c is obtained. Finally, given a string S, we call the CGR map x^S the *direct CGR index*, the CGR map x^{S^r} the *reverse CGR index*, the CGR map $x^{S^{rc}}$ the *reverse-complement CGR index*, and the CGR map x^{S^c} the *complement CGR index*.

3 Exact string matching

3.1 Finding all palindromes

A nucleotide sequence S is said to be a *(complemented) palindrome* if it is equal to its reverse complement. In this case, the first half of S converted to its reverse complement is equal to the second half of S. If the two halves of the palindrome are not adjacent it is said to be a *separated palindrome*. Moreover, the palindrome is said to have a *radius* correspondent to the size of each half. Palindromic sequences play an important role in molecular biology. They are, for instance, specifically recognized by many restriction endonucleases [3] and usually

associated with methylation sites [4].

The problem of finding all palindromes focus on uncovering all maximal complemented palindromes from a nucleotide sequence. By maximal it means that only the larger palindrome is extracted. For instance, the sequence TTATAA has a palindrome of radius 1 (AT), a palindrome of radius 2 (TATA), and a palindrome of radius 3 (TTATAA), but only the latter is maximal.

Finding all maximal palindromes require two CGR indexes: the reverse CGR index x^{S^r} and the complement CGR index x^{S^c}. Algorithm 1 presents the solution to this problem. The idea is to cover the string with LCE queries for the position pair $(\ell+1, N-\ell+1)$ in S and S^{rc}, respectively, with $1 \leq \ell \leq N-1$. Indeed, ignoring the complement issue, the position ℓ in S corresponds to the position $N-\ell+1$ in S^{rc}, since S^{rc} is reversed. So, a forward extension in S^{rc} from position $N-\ell+1$ corresponds to a backward extension from ℓ in S. It is clear now that a maximal palindrome is found by doing two extensions: (i) a forward extension from position $\ell+1$ in S; and (ii) a backward extension from position ℓ in S, which is the same as a forward extension from position $N-\ell+1$ in S^{rc}. If these extensions are given by $k > 0$, a maximal palindrome exists at position $k-\ell$ in S with radius k.

Algorithm 1 All exact maximal palindromes of S

ExactMaximalPalindromes(reverse CGR index x^{S^r}, complement CGR index x^{S^c})
1. for (ℓ from 1 to $N-1$)
2. let $k = \text{LCE}(x^{S^r}_{N-\ell}, x^{S^c}_{\ell})$ //longest common prefix of $S[\ell+1..]$ and $S^{rc}[N-\ell+1, ..]$
3. if ($k > 0$) there is a palindrome of radius k at position $\ell - k$ of S

Applying the alluded extensions for all feasible values of ℓ, corresponding to all positions in S, means that in $O(N)$ time we are able to find all maximal palindromes in S, as each of the $O(N)$ extensions queries are solved in constant time.

The other variant concerning separated palindromes can also be solved in linear time if there is a fixed gap between the two halves of the palindrome. The algorithm is a trivial modification of the previous one. Considering that a gap of size d is allowed the only modification would be in Step 2 where the LCE should be solved for position pairs $(\ell+1, N-\ell+d+1)$ in S and S^{rc}, respectively.

3.2 Finding all tandem repeats

Another major application that benefits from constant-time LCE queries is finding all tandem repeats in a nucleotide sequence. *Tandem repeats* are adjacent copies of a same DNA subsequence. They usually consist of short copies of 2-6 base pairs in length repeated throughout all eukaryotic genomes. Tandem repeats often represent important control sequences, such as upstream promoter sequences [5]. In addition, they have been used in forensic science for determining parentage [1].

The problem entailing tandem repeats focus in locating all tandem repeats in a nucleotide sequence. For instance, the sequence $TTATTA$ has a tandem repeat TT of size 2 and a tandem repeat $TTATTA$ of size 6, both starting in the first position of the nucleotide sequence. It also contains a tandem repeat TT of size 2 at position 4.

Without loss of generality, and for the sake of simplicity, we will focus our attention to tandem repeats with two copies only. The algorithm to find for all tandem repeats is a divide and conquer algorithm that recursively break down the problem of finding tandem repeats into four sub-problems, until these become simple enough to be solved directly. The four sub-problems are of two different types. To understand why first notice that a tandem repeat may occur either in the first or in the second half of the string, or in the middle of it. In the latter, either the first or the second copy of the tandem repeat may span the middle position of the string. Therefore, the idea is to recursively break down the original string by asking for tandem repeats that appear currently in the first or in the second half of the string, but that do not span its middle position, until we reach a string of minimum size 2. This is the sub-problem of type I. In subsequent processing, these tandem repeats will actually occur in the middle of some substring attained by recursively breaking down the string in two halves. Finding for tandem repeats in the middle positions is the type II sub-problem. This sub-problem is indeed the core of the algorithm.

The algorithm to find all exact tandem repeats is given in Algorithm 2, and the sub-problem of type II is addressed in Algorithm 3. The algorithm receives two standard CGR indexes, needed to compute two distinct LCE queries (only in Algorithm 3), and two absolute positions in S, denoted by h_1 and h_2. Comprehensibly, the first time this algorithm is called $h_1 = 1$ and $h_2 = N$. Algorithm 2 starts by checking if $|S[h_1..h_2]|$ is greater than 1 in Step 2 as no tandem repeats of size less than 2 are possible. If so, it computes the midposition of

$S[h_1..h_2]$, denoted by h, in Step 3. This midposition is then used to break down the problem into finding tandem repeats in the first (Step 4) or in the second (Step 5) half of $S[h_1..h_2]$. This undertakes sub-problems of type I. Steps 6–8 address sub-problems of type II. Therein, all possible manners of finding a tandem repeat that spans the midposition h may be checked. Taking into account that the size of a tandem repeat that spans h may vary from 1 to at most h, two checks are made in order to verify if there is a tandem repeat whose first copy spans position h and, moreover, if there is a tandem repeat whose second copy spans position h.

Algorithm 2 All exact tandem repeats of S

ExactTandemRepeats(direct CGR index x^S, reverse CGR index x^{S^r}, start position h_1, end position h_2)

1. let $\ell = h_2 - h_1 + 1$
2. if ($\ell > 1$)
3. let $h = \lfloor \frac{\ell}{2} \rfloor$
4. call ExactTandemRepeats(x^S, x^{S^r}, h_1, $h_1 + h - 1$) //search for tandem repeat in first half of $S[h_1..h_2]$
5. call ExactTandemRepeats(x^S, x^{S^r}, $h_1 + h$, h_2) //search for tandem repeat in second half of $S[h_1..h_2]$
6. for (l from 1 to h)
7. if ($h + l \leq N$) call ETR–SP($x^S, x^{S^r}, h, h + l, l$) //check if there is a tandem repeat of length $2l$ whose first copy spans position h of S
8. if ($h - l \geq 1$) call ETR–SP($x^S, x^{S^r}, h - l, h, l$) //check if there is a tandem repeat of length $2l$ whose second copy spans position h of S

Algorithm 3 deserves a deeper explanation. Finding a tandem repeat of a given size l that spans some position in the string (h or q depending on how it was called in Step 7 or Step 8 of Algorithm 2) is done in constant time. It resumes to perform two LCE queries and analyze the overlap window of their result. If there is an overlap inside the left and right positions (h and q, respectively) then the overlapped positions correspond to possible midpositions of tandem repeats of size $2l$ (Step 3).

Algorithm 3 Sub-procedure of all exact tandem repeats of S

ETR–SP(direct CGR index x^S, reverse CGR index x^{S^r}, left position h, right position q, size l)

1. let $l_1 = \text{LCE}(x^{S^r}_{N-h+1}, x^{S^r}_{N-q+1})$ //longest common prefix of $S[h..]$ and $S[q..]$
2. let $l_2 = \text{LCE}(x^{S}_{N-h+2}, x^{S}_{N-q+2})$ //longest common prefix of $S^r[h-1..]$ and $S^r[q-1..]$
3. if ($l_1 + l_2 \geq l$ and $l_1 \geq 1$ and $l_2 \geq 1$) there is a tandem repeat of size $2l$ whose first copy spans position h; return the pair $\langle \max(h - l_2, h - l + 1), \ldots, \min(h + l_1 - l, h), 2l \rangle$ where the first component is the set of starting positions of $2l$-size tandem repeats of S (whose first copy spans position h)

The time complexity of Algorithm 2 is $O(N \log N + z)$ where z is the number of tandem

repeats in S. The $N \log N$ term comes from the number of extensions executed for a string of size N. This number is given by $T(N) = 2T(\frac{N}{2}) + 2N$, hence, $T(N) = O(N \log N)$. Independently from the number of extensions performed, the output size is z. As over the entire execution of Algorithm 2 no tandem repeat is reported twice, the total time to output all tandem repeats is $O(z)$. With this we conclude time complexity analysis of Algorithm 2.

4 Approximate string matching

4.1 Finding all k-mismatches of a pattern

The *k-mismatch* problem is an approximate matching problem where k mismatches are allowed. Approximate matching is a mandatory task in molecular biology as DNA sequence patterns are usually described in an approximate way. This makes way for more relaxed versions of palindrome and other repeat problems.

Given a string S, a pattern P, and a fixed number of mismatches k, the k-mismatch problem finds all matches of P in S with at most k mismatches. The general idea of the algorithm is to perform up to $k+1$ exact matches intercalated with up to k mismatches. Each exact match is answered with a LCE query.

Assuming that S has size N and P has size M, the pseudo-code of the k-mismatch problem is given in Algorithm 4. It covers all possible positions in S (Step 2) to check for the approximate match of P in $S[i..i+M-1]$ (Steps 3–7), with $1 \leq i \leq N-M+1$. Concerning variables, *count* stores the number of mismatches found in the inner while cycle (being reset to zero by the outer while cycle). Moreover, variable j saves the number of nucleotides already inspected in P and i' the corresponding position in S. If the end of P was not yet reached in Step 6, these variables are updated in Step 7 to comprise the extension and the mismatch. Moreover, the number of mismatches are also updated within variable *count*. However, if in Step 6 the end of P was already reached, then P is a k-mismatch of $S[i..i+M-1]$. Actually, P is a *count*-mismatch of $S[i..i+M-1]$ as only *count* mismatches were added up by the inner while cycle (observe that $count \leq k$ at Step 7). If P does not occur in $S[i..i+M-1]$ with up to k-mismatches then condition in Step 6 is always evaluated to false and a $count = k+1$ will be reached. In that case the inner while cycle fails and a new position $i+1$ in S is attempted by the outer while cycle.

Algorithm 4 All k-mismatches of P in S

kMismatches(reverse CRG index x^{S^r}, pattern P, number of mismatches k)

1. let x^{P^r} be the reverse CGR index of P
2. for (i from 1 to $N - M + 1$)
3. let $count = 0$, $j = 1$ and $i' = i$
4. while ($count \leq k$) do
5. let $\ell = \mathsf{LCE}(x^{P^r}_{M-j+1}, x^{S^r}_{N-i'+1})$//longest common prefix of $P[j..]$ and $S[i'..]$
6. if ($j + \ell = M + 1$) a $count$-mismatch of P occurs in S starting at i; break
7. let $count = count + 1$, $j = j + \ell + 1$ and $i' = i' + \ell + 1$

The k-mismatch problem can be solved in $O(kN)$ time. Indeed, each match operation in Steps 3–7 takes at most $O(k)$ time, and the scan of the string in Step 2 takes $O(N)$ time. The computation of the reverse index of P in Step 7 is accomplished in time proportional to the patter size, that is, $O(k)$ time. Therefore, the overall complexity of Algorithm 4 is given by $O(k + kN) = O(kN)$.

4.2 Finding all k-mismatch palindromes

A simple variant of the k-mismatch algorithm can be used to solve the problem of all k-mismatch palindromes. The pseudo-code to solve this problem is presented in Algorithm 5.

Algorithm 5 All k-mismatch maximal palindromes in S

kMismatchMaximalPalindromes(reverse CGR index x^{S^r}, complement CGR index x^{S^c}, number of mismatches k)

1. let S^{rc} be the complement of S^r
2. for (i from 1 to $N - 1$)
3. let $count = 0$, $i' = i$ and $e = 0$
4. while ($count \leq k$) do
5. let $\ell = \mathsf{LCE}(x^{S^r}_{N-i'}, x^{S^c}_{i'})$ //longest common prefix of $S[i' + 1..]$ and $S^{rc}[N - i' + 1..]$
6. if ($count = k$) a $count$-mismatch palindrome of radius $e + \ell$ occurs in S centered at i; break
7. let $count = count + 1$, $i' = i' + \ell + 1$ and $e = e + \ell + 1$

4.3 Finding all k-mismatch tandem repeats

A variant of the k-mismatch algorithm can also be used to solve the all k-mismatch tandem repeats. The modus operandi for all k-mismatch tandem repeats [2] is also coincident with the same problem without mismatches, but the algorithm is more tangled. The only change

from the exact case is in the LS–procedure presented in Algorithm 7.

Algorithm 6 All k-mismatch tandem repeats in S

kMismatchTandemReapeats(direct CGR index x^S, reverse CGR index x^{S^r}, string S, left position h, right position q, number of mismatches k)

1. let $count = \ell_1 = \ell_2 = 0$ and $p_1 = \emptyset$ and $p_2 = \emptyset$
2. while ($count \leq k$)
3. let $l_1 =$ LCE$(x^{S^r}_{N-h+1}, x^{S^r}_{N-q+1})$ //longest common prefix of $S[h..]$ and $S[q..]$
4. let $l_2 =$ LCE$(x^{S}_{N-h+2}, x^{S}_{N-q+2})$ //longest common prefix of $S^r[h-1..]$ and $S^r[q-1..]$
5. let $\ell_1 = \ell_1 + l_1$ and $\ell_2 = \ell_2 + l_2$
6. if ($h + l_1 \leq q$) let $p_1 = p_1 \cup \{h + l_1\}$
7. if ($q - l_2 \geq h$) let $p_2 = p_2 \cup \{q - l_2\}$
8. if ($count = k$)
9. if ($\ell_1 + \ell_2 + 2k \geq l$ and $\ell_1 \geq k+1$ and $\ell_2 \geq k+1$) there is a k-mismatch tandem repeat of size $2l$ whose midposition starts in a point in $M = k$TR–Positions$(p_1 \cup p_2, h, q, k)$; return the pair $\langle m_i - l, 2l\rangle_{m_i \in M}$ where the first component is the set of starting positions of $2l$-size tandem repeats of S
10. break
11. let $count = count + 1$, $h = h - l_2 - 1$ and $q = q + l_1 + 1$

Given a list p (of positions) the i'th element of p is denoted by $p[[i]]$. The first element of the list p is at index 1, therefore, $1 \leq i \leq length(p)$, where $length(p)$ denotes the number of elements in p.

Algorithm 7 Auxiliary procedure of all k-mismatch tandem repeats in S

kTR-positions(list of positions p, left position h, right position q, number of mismatches k)

1. let $r = \emptyset$
2. let p be ordered in increasing order and prepend h to p and append q to p
3. for (i from 2 to $length(p) - 1$)
4. let l be the number of mismatches from h to $p[[i]]$ given by forward extensions (Step 3, AllkMismatchTandemReapeats procedure)
5. let r be the number of mismatches from q to $p[[i]]$ given by reverse extensions (Step 4, AllkMismatchTandemReapeats procedure)
6. if ($l + r \leq k$) positions in the interval $[p[[i-1]], p[[i]])$ are possible midpositions of the tandem repeat, so let $r = r \cup \{p[[i-1]], \ldots, p[[i]]\}$
7. return r

References

[1] A. L. Baumstark, B. Budowle, D. A. Defenbaugh, J. B. Smerick, K. M. Keys, and T. R. Moretti. Validation of short tandem repeats (STRs) for forensic usage: Performance testing of fluorescent multiplex STR systems and analysis of authentic and simulated forensic samples. *Journal of Forensic Sciences*, 46(3):647–660, 1999.

[2] Gad M. Landau, Jeanette P. Schmidt, and Dina Sokol. An algorithm for approximate tandem repeats. *Journal of Computational Biology*, 8(1):1–18, 2001.

[3] A. Pingoud and A. Jeltsch. Structure and function of type II restriction endonucleases. *Nucleic Acids Research*, 29(18):3705–3727, September 2001.

[4] N. O. Reich and M. J. Danzitz. Non-additivity of sequence-specific enzyme-DNA interactions in the EcoRI DNA methyltransferase. *Nucleic Acids Research*, 19(23):6587–6594, December 1991.

[5] Marcelo D. Vinces, Matthieu Legendre, Marina Caldara, Masaki Hagihara, and Kevin J. Verstrepen. Unstable tandem repeats in promoters confer transcriptional evolvability. *Science*, 324(5931):1213–1216, May 2009.
